# Comparative Analysis of Three Bradyrhizobium diazoefficiens Genomes Show Specific Mutations Acquired during Selection for a Higher Motility Phenotype and Adaption to Laboratory Conditions

**DOI:** 10.1128/Spectrum.00569-21

**Published:** 2021-11-17

**Authors:** Mauricio J. Lozano, Miguel Redondo-Nieto, Daniel Garrido-Sanz, Elías Mongiardini, J. Ignacio Quelas, Florencia Mengucci, Carolina Dardis, Aníbal Lodeiro, M. Julia Althabegoiti

**Affiliations:** a Instituto de Biotecnología y Biología Molecular (IBBM), Facultad de Ciencias Exactas, UNLP-CONICET, La Plata, Argentina; b Departamento de Biología, Facultad de Ciencias, Universidad Autónoma de Madrid, Madrid, Spain; c Laboratorio de Genética, Facultad de Ciencias Agrarias y Forestales, Universidad Nacional La Plata (UNLP), La Plata, Argentina; Dublin City University

**Keywords:** laboratory adaptation, single nucleotide polymorphism, swimming motility, comparative genomics

## Abstract

Microbial genomes are being extensively studied using next-generation sequencing technologies in order to understand the changes that occur under different selection regimes. In this work, the number and type of mutations that have occurred in three Bradyrhizobium diazoefficiens USDA 110^T^ strains under laboratory conditions and during selection for a more motile phenotypic variant were analyzed. Most of the mutations found in both processes consisted of single nucleotide polymorphisms, single nucleotide deletions or insertions. In the case of adaptation to laboratory conditions, half of the changes occurred within intergenic regions, and around 80% were insertions. When the more motile phenotypic variant was evaluated, eight single nucleotide polymorphisms and an 11-bp deletion were found, although none of them was directly related to known motility or chemotaxis genes. Two mutants were constructed to evaluate the 11-bp deletion affecting the alpha subunit of 2-oxoacid:acceptor oxidoreductase (AAV28_RS30705-blr6743). The results showed that this single deletion was not responsible for the enhanced motility phenotype.

**IMPORTANCE** The genetic and genomic changes that occur under laboratory conditions in Bradyrhizobium diazoefficiens genomes remain poorly studied. Only a few genome sequences of this important nitrogen-fixing species are available, and there are no genome-wide comparative analyses of related strains. In the present work, we sequenced and compared the genomes of strains derived from a parent strain, *B. diazoefficiens* USDA 110, that has undergone processes of repeated culture in the laboratory environment, or phenotypic selection toward antibiotic resistance and enhanced motility. Our results represent the first analysis in *B. diazoefficiens* that provides insights into the specific mutations that are acquired during these processes.

## INTRODUCTION

Microbes are well suited to be studied under laboratory conditions due to several features, including their ease of growth and storage, short generation times, and discrete genome sizes. The types of genetic changes and the frequencies with which they accumulate in evolving populations over time guides a general understanding of any biological system (1). These genetic changes occur at random genomic positions and are selected either by neutral selection during repeated growth under laboratory conditions or by positive selection (e.g., applying a selective pressure such as antibiotic resistance, or artificially selecting strains with higher motility, among others). During long periods of repeated growth in rich media, genetic changes are accumulated in a process sometimes called the “domestication” of strains (2–4), while genetic changes that occur during the selection of variants with a desired phenotype are guided by a selective pressure (5–7) imposed by the researcher. Genetic studies on the number and type of mutations acquired during repeated growth or the selection of phenotypic variants have been focused so far on a limited number of species. For example, the genetic changes accumulated over 20,000 Escherichia coli generations in a long-term experiment (8) show that discrete duplication events can lead to novel phenotypic traits (9). However, the genetic changes that drive the selection of other phenotypic traits in different species remain unexplored.

Bradyrhizobium diazoefficiens is a symbiotic bacterium of the order *Rhizobiales* that can nodulate soybean to fix atmospheric nitrogen. There are no previous comparative genomic studies on this species, neither under repeated growth in the laboratory nor during the selection of phenotypic variants. *B. diazoefficiens* USDA 110^T^ was first sequenced in 2002 by Kaneko et al. (10) (GenBank accession number NC_004463). In 2016, Davis-Richardson et al. deposited a new sequence of *B. diazoefficiens* USDA 110 at NCBI (NZ_CP011360) as a product of a genome-wide analysis of DNA methylation (11).

In the current work, we sequenced three *B. diazoefficiens* strains from our laboratory: our own stock of *B. diazoefficiens* USDA 110 (LP USDA 110), LP 3004, and LP 3008. The original USDA 110 stock that gave origin to LP USDA 110 was received from the U.S. Department of Agriculture at least 35 years ago. LP 3004 is a spontaneous streptomycin-resistant strain obtained from LP USDA 110 in 1994 in our laboratory. LP 3008 is a higher-motility strain derived from LP 3004 by recurrent selection from the external rings of swimming halos and reinoculation into new plates after 13 rounds of selection, obtained in 2003 (12, 13). All strains were routinely used in the laboratory and maintained as frozen stocks at −80°C that were renewed every 5 years. We analyzed the genetic changes that accumulated in LP USDA 110 and LP 3004 under laboratory culture conditions by comparing their genome sequences with the reference sequence of Davis-Richardson and colleagues (11). Additionally, the genome of the more motile LP 3008 strain was compared against its parent strain (LP 3004) in order to identify the mutations responsible for its enhanced motility.

## RESULTS

The sequence of *B. diazoefficiens* USDA 110 obtained by Davis-Richardson et al. (11) (hereafter, DR USDA 110) was used in this work as the reference genome sequence. The three genome sequences of *B. diazoefficiens* obtained in this work were compared with that sequence, and a new comparison between DR USDA 110 and that published by Kaneko et al. (10) (hereafter, K USDA 110) was also conducted.

### Genetic changes in *B. diazoefficiens* LP USDA 110 and LP 3004 compared with the reference sequence DR USDA 110.

**FIG 1:**
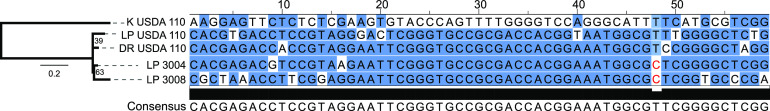
Maximum-likelihood phylogenetic tree generated using the SNPs of the core genome. Snippy-core was used to generate the alignment of the core genome SNPs between the sequences of the reference strains USDA 110, K USDA 110 (Kaneko et al.) (10), and DR USDA 110 (Davis-Richardson et al.) (11) and the draft genome sequences of LP USDA 110, LP 3004, and LP 3008. The shading corresponds to the conservation level. The letters in red indicate the mutation on the *rpsL* gene of LP 3004 and LP 3008 which provides streptomycin resistance. The numbers at the nodes indicate bootstrap support.

**FIG 2:**
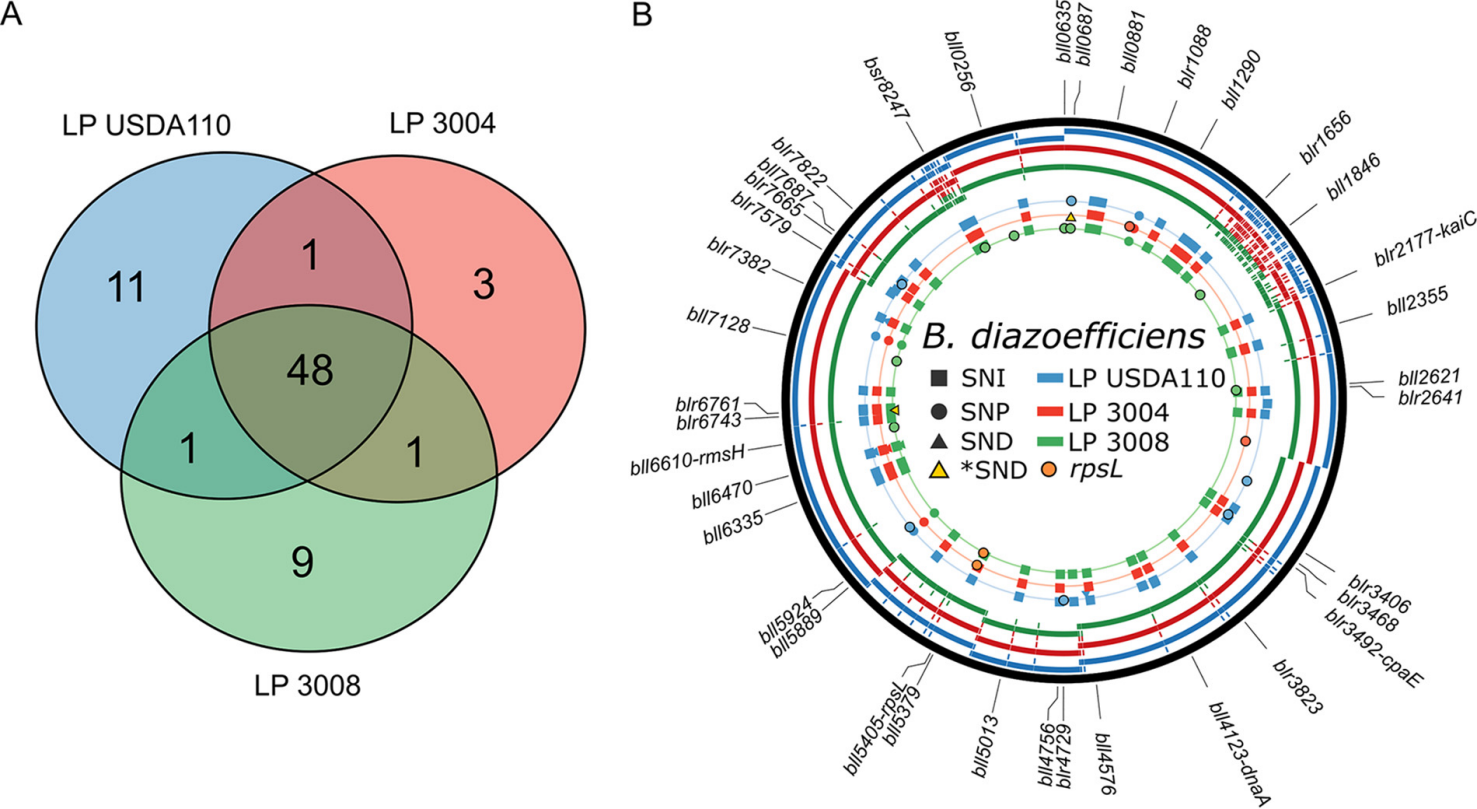
Comparison of the draft genome sequences of LP USDA 110, LP 3004, and LP 3008. (A) Venn diagram of the DNA polymorphisms found comparing the reference strain DR USDA 110 and each of the strains sequenced, using Snippy software. The overlapping areas show the number polymorphisms shared between the three sequenced strains of this study. (B) Genome comparison between LP USDA 110, LP 3004, and LP 3008 and the reference strain DR USDA 110. The innermost to outermost circles indicate the position of the single nucleotide insertions (SNIs, squares), single nucleotide polymorphisms (SNPs, circles), and single nucleotide deletions (SNDs, triangles). Multiple nucleotide deletions are represented by yellow triangles. The genome assemblies were mapped against the reference genome (black line). The locus tag (old annotation) of the genes presenting a polymorphism is shown. The colors indicate the strains as follows: blue, LP USDA 110; red, LP 3004; and green, LP 3008. The orange circles represent the SNPs on the *rpsL* gene.

We employed Snippy v3.1 software (14) to identify variations between both USDA 110 genome sequences available at NCBI. A total of 125 polymorphisms were found (see Table S1 at https://ibbm.conicet.gov.ar/althabegoiti/table_s1/); however, some of them could be errors that arose in the Davis-Richardson sequencing and assembly (PacBio), since our three newly sequenced strains and K USDA 110 shared 48 of these 125 mutations. In addition, we detected 72 mutations exclusive of K USDA 110 and consider that they also could be sequencing errors that arose because of the low quality of the methods employed in 2002. Moreover, we compared the previously deposited genome sequences of K USDA 110 and DR USDA 110 with those of LP USDA 110, LP 3004, and LP 3008 and performed a phylogenetic analysis of the core single nucleotide polymorphisms (SNPs). The results show that our strains are more similar to the DR USDA 110 sequence (Fig. 1); therefore, we decided to continue using this sequence as the reference. Also, our results provide evidence that most contig boundaries are common among our strains, which might indicate that there were few or no variations in the mobile element positions (Fig. 2B), which is in agreement with nearly clonal strains where no large genomic rearrangements have occurred.

The variant-calling analysis against the DR USDA 110 genome resulted in 61 mutations for LP USDA 110 and 53 for LP 3004, most of them implying a 1-bp change. In total, 49 mutations were common to both LP USDA 110 and LP 3004. Of the 61 mutations found in LP USDA 110, including 9 single nucleotide polymorphisms (SNPs), 3 single nucleotide deletions (SNDs), and 49 single nucleotide insertions (SNIs), 45% were located within intergenic sequences, and 11 were only found in LP USDA 110 (Fig. 2A). The effect of each mutation is specified in Table S1 (https://ibbm.conicet.gov.ar/althabegoiti/table_s1/). On the other hand, of the 53 changes in LP 3004 (6 SNPs, 2 deletions, and 45 SNIs), 3 are present only in this strain, 1 is shared with LP USDA 110, and 1 is shared with LP 3008 (Fig. 2A). The three mutations exclusive to LP 3004 are a 12-bp deletion and two SNPs. The deletion is particular since it generates an in-frame deletion at position 52554, locus AAV28_RS00270-bll0687 (new annotation-old annotation). The SNPs are located at positions 520764 and 2595743 and correspond to a synonymous mutation in the locus AAV28_RS02285-blr1088 and an alteration in an intergenic region, respectively. The SNP at position 5260582 is common to LP 3008 and is located in the *rpsL* gene (AAV28_RS023835-bll5405). This SNP is crucial because this gene encodes the unique 30S ribosomal protein S12 (15–18), and this mutation allows the ribosome to function in the presence of streptomycin, explaining the antibiotic-resistant phenotype displayed by this strain. The SNP within *rpsL* of LP 3004 and LP 3008 produces a conservative change from lysine (K) to arginine (R) in position 87 of the protein.

### Genetic changes between *B. diazoefficiens* LP 3008 and LP 3004.

LP 3008 was obtained from LP 3004 by the selection of phenotypic variants with increased swimming motility in semisolid agar and a derepressed expression of lateral flagella in Götz mannitol medium (12). Interestingly, aside from the mutations shared by both strains, 9 additional mutations unique to LP 3008 were found (Table 1, Fig. 2A and B). These genetic changes could be attributed either to the divergence under laboratory conditions of each strain (as observed between LP USDA 110 and the DR USDA 110 reference genome) or to the higher ability of LP 3008 to swim in semisolid agar medium. Regarding LP 3008, none of its mutations were located in flagellar or chemotaxis genes. Among the mutations, one of them corresponds to an 11-bp deletion that generates a frameshift in the alpha subunit of the 2-oxoacid:acceptor oxidoreductase (AAV28_RS30705-blr6743) gene, involved in the central carbohydrate metabolism pathways in *B. diazoefficiens*, according to the KEGG PATHWAY database (19). Blr6743 and Blr6744 are alpha and beta subunits of an oxidoreductase that uses coenzyme A (CoA) and ferredoxin as cofactors and is responsible for the oxidation of pyruvate to acetyl-CoA or 2-oxoglutarate to succinyl-CoA. Furthermore, Blr6743 and Blr6744 were found expressed in a proteomic study of *B. diazoefficiens* LP USDA 110, both in the cytoplasmic and membrane fractions (20). Thus, the deletion in the alpha subunit of the enzyme could cause a metabolic alteration in LP 3008, triggering its enhanced swimming phenotype.

**TABLE 1:** Mutations found in Bradyrhizobium diazoefficiens LP 3008 in comparison with its parental strain, LP 3004^*a*^

Locus tag	Old locus tag	Product	Genome position	Type	Change	Strand	NT position	AA position	Effect
AAV28_RS00010	bll0635	Transcription termination factor Rho	2257	SNP	GxT	**−**	1093/1266	365/421	miss_var, C > A, Arg365Ser
AAV28_RS00270	bll0687	Methylated-DNA−[protein]-cysteine *S*-methyltransferase	52554	SNP	GxA	**−**	607/897	203/298	stop_gained, C > T, Gln203Stop
AAV28_RS05940	bll1846	Hypothetical protein	1319462	SNP	CxT	**−**	545/1068	182/355	miss_var, G > A, Arg182Lys
AAV28_RS09610	bll2621	l,d-Transpeptidase	2189956	SNP	TxG	**−**	224/687	75/228	miss_var, A > C, Asp75Ala
AAV28_RS30020	bll6610	16S rRNA (cytosine^1402^-N^4^)-methyltransferase RsmH	6604916	SNP	GxT	**−**	331/1164	111/387	miss_var, C > A, Arg111Ser
AAV28_RS30705	blr6743	2-Oxoacid:acceptor oxidoreductase subunit alpha	6746974	del	−11 bp	**+**	522/1923	174/640	frameshift_var, 522_532del
AAV28_RS32660	bll7128	Cell division protein	7163641	SNP	TxC	**−**	2627/2967	876/988	miss_var, A > G, Lys876Arg
	Intergenic		8413344	SNP	GxA				
AAV28_RS39765	bll0256	AraC family transcriptional regulator	8677994	SNP	AxG	**−**	473/966	158/321	miss_var, T > C, Ile158Thr

a Change (NT in LP 3004 × NT in LP 3008); NT pos, nucleotide position; AA, amino acid position; effect, the consequence of the variant (miss_var, missense variation, NT change and AA change).

The remaining eight mutations unique to LP 3008 are SNPs. The SNP at position 52242, locus AAV28_RS00270-bll0687, generates a stop codon in this gene. Bll0687 is a far homolog of the E. coli *ada* gene (33% sequence identity, 83% coverage), encoding a putative methylated-DNA–[protein]-cysteine *S*-methyltransferase. Bll0687 presents two conserved domains (21), an AraC-type DNA-binding domain (positions 8 to 124) and an AdaB domain (O6-methylguanine-DNA–[protein]-cysteine methyltransferase, positions 130 to 293). It has been shown that these two Ada domains in E. coli can function independently, activating the transcriptional regulator Ada to stimulate the Ada regulon (22). This response has been deeply studied in E. coli, where the expression of four genes, *ada*, *alkB*, *alkA*, and *aidB*, was increased and induced alkylation resistance (23). Homologs for all these genes are present in *B. diazoefficiens*. In LP 3008, the acquired stop codon likely results in a truncated Ada protein, disturbing its methyltransferase domain (Table 1). Although Bll0687 was not found in the previously mentioned proteomic study (20), it may be a consequence of the bacterial growth conditions used or a putative low protein concentration. Additionally, transposon insertion mutants for homologs of the E. coli Ada protein in Ensifer meliloti (24, 25) did not show a distinctive phenotype.

Two other interesting SNP mutations found uniquely in LP 3008 that could result in global changes are those found at position 2257, locus AAV28_RS00010-bll0635 (*rho*), and at position 6604916, locus AAV28_RS30020-bll6610 (*mraW*). Both mutations result in missense variations involving a replacement of arginine (R) with serine (S). In the transcription termination factor Rho, the change does not affect any functional domain, as the R365S substitution is located in a low-conserved and exposed position among orthologous sequences, according to our analysis using ConSurf (26). Alterations near position 365 did not show any evident phenotype in a previous study (27). On the other hand, MraW is a methyltransferase described in E. coli, responsible for the methylation of the 1402 cytosine (N^4^ methylation) of the 16S rRNA (23, 28). 16S rRNAs are highly conserved and have some positions with special modifications; for example, m4Cm1402 plays a role in adapting the P site of the ribosome to enhance the decoding fidelity (23, 29). The R111 of MraW is moderately conserved (ConSurf score, 7), which implies that the R111S mutation present in LP 3008 could be relevant for the observed phenotype. Although it has been noted that in E. coli, the 16S rRNA modifications are not needed for the functional activity of *in vitro*-reconstituted ribosomes (28), several studies indicate that MraW could be important for bacterial fitness, stress tolerance, and virulence and might be essential in some bacteria (24, 29–31). Additionally, a recent report shows a new function for E. coli MraW as a DNA methylase with a wider role, affecting different flagellar genes and promoters and therefore implicated in motility (32). The remaining five LP 3008 specific mutations are all missense variants. As indicated in Table 1, one SNP is located in locus AAV28_RS05940-bll1846, which encodes a hypothetical protein without characterized domains. This SNP occurs in variable positions (ConSurf) and replaces R with K, suggesting that it could be a conservative mutation. Also, this protein is only present in *Bradyrhizobiaceae*, with some distant homologs in species from the genera *Ensifer*, *Mesorhizobium*, and *Microvirga*. The second SNP is situated at position 2189956, locus AAV28_RS09610-bll2621. Bll2621 is homologous to an l,d-transpeptidase which is involved in peptidoglycan biosynthesis in E. coli (33). The SNP occurs within a conserved region that affects the YkuD domain, indicating that this protein function might be altered, although transposon insertion mutants in the homologous protein SMc04338 of *E. meliloti* (45% identity, 80% coverage) do not have a strong phenotype (24, 25). The third SNP of this group is located at position 7163641, locus AAV28_RS32660–bll7128, whose product is an eight-transmembrane domain protein annotated as a cell division protein; however, the mutation occurred in a variable position (ConSurf) of the C-terminal region, in which no functional domains were annotated. Additionally, some bll7128 orthologs in the genus *Bradyrhizobium* presented a conservative K–R substitution. The fourth SNP of this set is positioned within an intergenic region, position 8413344, between genes AAV28_RS38515–blr8303 and AAV28_RS38520–blr8304, not affecting their promoters and near the 3′ region of both mRNAs. Finally, the fifth SNP of this set is found at position 8677994, locus AAV28_RS39765-bll0256, which encodes an AraC family transcriptional regulator and apparently does not affect the unique helix-turn-helix (HTH) domain of the protein. Yet this amino acid appears at a conserved position within homologous proteins according to ConSurf. However, none of the similar proteins of *E. meliloti* 1021 (PSI-BLAST search, ≥70% query coverage; E value, <1.10^−4^) produced an altered phenotype when mutated by transposon insertion (24, 25).

### Blr6743 is not responsible for the enhanced motility phenotype of *B. diazoefficiens* LP 3008.

LP 3008 has two phenotypic characteristics, a higher motility trait in 0.3% (wt/vol) agar-containing Götz medium (34) compared with the wild type (wt) and the expression of the lateral flagellum (composed of Laf proteins) in the same liquid medium, while LP 3004 only expresses the subpolar flagellum (composed of FliC proteins). Nine mutations were found in LP 3008, one of them consisting of an 11-bp deletion. The deletion occurs at amino acid 174/640 of the 2-oxoacid:acceptor oxidoreductase alpha subunit (Blr6743), resulting in a frameshift in the polypeptide. The protein Blr6743 seems to catalyze an oxidative decarboxylation process, with ferredoxin being the cognate cofactor. This protein has two annotated domains, the first one (Pfam: PF01558) between amino acids 47 and 213. The PF01558 family domain includes a region of the large protein pyruvate-flavodoxin oxidoreductase and the whole pyruvate ferredoxin oxidoreductase gamma subunit protein. The second domain (Pfam: PF01855), amino acids 248 to 412, includes the N-terminal structural domain of the pyruvate ferredoxin oxidoreductase that binds thiamine diphosphate. Therefore, Blr6743 could be involved in the oxidation of pyruvate to acetyl-CoA or 2-oxoglutarate to succinyl-CoA. Since both domains were affected by the frameshift, the activity of this protein could be affected and explain the observed phenotype. Nonetheless, the process by which the cell drives the overexpression of the lateral flagellum, which produces an enhanced motility phenotype in swimming plates, is yet unknown (12).

**FIG 3:**
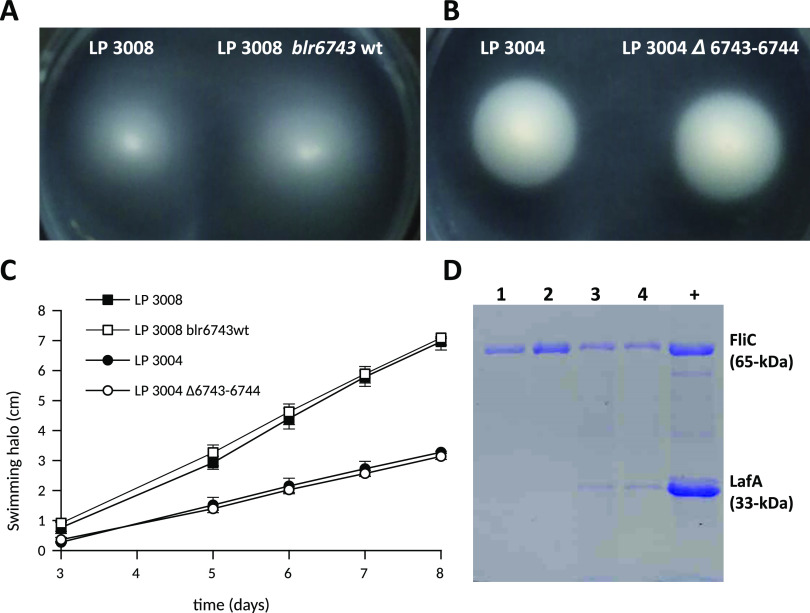
Phenotypic characterization of *B. diazoefficiens* blr6743 mutants in different genomic backgrounds. Swimming plates in 0.3% (wt/vol) agar containing Götz medium incubated at 28°C were performed for LP 3008 and LP 3008 carrying blr6743 wt (A) and LP 3004 and LP 3004 *Δ6743-6744* (B). (C) Swimming performance of LP 3008, LP3008 blr6743 wt, LP 3004, and LP 3004 *Δ6743-6744*. Cells of each of these strains were inoculated individually onto a plate, and the diameter of the halo was measured at the indicated times. Four technical replicates of each strain were performed. (D) SDS-PAGE of extracellular *B. diazoefficiens* proteins of LP 3004 (lane 1), LP 3004 *Δ6743-6744* (lane 2), LP 3008 (lane 3), LP 3008 blr6743 wt (lane 4), and a positive control (+) with both flagellin proteins of *B. diazoefficiens*. Each experiment was repeated twice.

We complemented the wild-type allele of blr6743 in the LP 3008 genomic background. We cloned 348 bp of the allele from LP 3004 into the suicide vector pK18*mobsacB* to obtain pK18*mobsacB*::Up_wt and transferred it to LP 3008, to obtain an LP 3008 blr6743 wt. However, the phenotype was not reversed with the complementation of the wild-type allele (Fig. 3A and C). As a second approach, we employed LP 3004 to perform an in-frame deletion of the blr6743 and blr6744 genes, which are separated by only 3 bp. We tested the flagellin production of strain LP 3004 *Δ6743-6744* (lacking the 2-oxoacid:acceptor oxidoreductase enzyme) in liquid cultures and its phenotype in swimming plates of Götz medium, but no changes were found in the lateral flagella production or motility, respectively (Fig. 3B and C).

## DISCUSSION

Here, we analyzed the genomic changes in closely related strains, all derivatives of *B. diazoefficiens* USDA 110 that underwent different selection processes. The results suggest that under laboratory culture conditions over a long time, most of the mutations consist of 1-bp SNDs, SNIs, or SNPs, with half of the SNPs located in noncoding regions and, apparently, without a particular phenotype (4, 35). In this process, SNIs are the most common mutations, accounting for 80% in LP USDA 110 and 85% in LP 3004, probably caused by errors in DNA replication generated by strand slippage, since they are, predominantly, an extra C or G in poliC or poliG sequences, respectively. Furthermore, they could be errors of the sequencing techniques in the homopolymer sequence. In particular, in LP 3004, the mutation in *rpsL* was selected by growing *B. diazoefficiens* LP USDA 110 in rich medium supplemented with streptomycin as the selective pressure (A. Lodeiro, unpublished data). On the other hand, when 13 rounds of selection to improve motility were performed, the number of total changes decreased, but most of them were located in coding regions. However, none of these mutations directly affected genes related to motility, whether structural or regulatory, nor in chemotaxis proteins, as occurred in seven evolved strains of E. coli obtained by a similar selection process. In that work, the authors found mutations in genes related to flagellar function, metabolism, and transport, among others (36). The number of mutations they found ranged from two to nine SNPs per sequenced strain, similar to our finding for *B. diazoefficiens* LP 3008. Conversely, two publications that performed and screened library mutant libraries of Salmonella enterica (37) and E. coli (38) found that higher motility phenotypes are due to alterations in different proteins that are not directly associated with motility.

The nine mutations found in our study, or combinations of them, are likely responsible for the high-motility phenotype. We initially thought that the most likely candidate was the deletion of Blr6743, the 2-oxoacid:acceptor oxidoreductase subunit alpha, since we hypothesized that this mutation could generate a metabolic change effect resulting in the observed phenotype. Nevertheless, after replacing the mutated gene with its wild-type version in the LP 3008 background, or deleting the genes blr6743 and 6744 in LP 3004, we could not observe any phenotypic change, at least with this single alteration.

On the other hand, some SNPs are less likely candidates, such as Bll7128, which presents a K → R substitution in a variable region that is also present in some Bll7128 orthologs within the genus *Bradyrhizobium*; Bll1846, where the SNP is R → K and occurs in a variable position, suggesting that the protein might be unaltered, or the SNP at the intergenic region. For the rest of the mutations, it is more difficult to hypothesize about their involvement in the observed phenotype. As an example, DNA alteration in AAV28_RS30020-bll6610 could lead to lower motility, indicating a possible indirect role of *mraW*. Recently, the role of MraW as DNA methylase has been described, with implications for motility (32), highlighting MraW as one of the most likely candidates to explain the high-motility phenotype of LP 3008. Xu et al. (32) found 336 genes and 219 promoters which showed a decrease of methylation in the *mraW* mutant, including 4 flagellar gene and promoter sequences. Additionally, they observed that MraW directly binds to the four flagellar gene sequences and identified a common motif in the differentially methylated regions of their promoters and coding sequences. However, we could not find the motifs with certainty in either the flagellar genes of E. coli or those of *B. diazoefficiens*.

## MATERIALS AND METHODS

### DNA extraction and sequencing.

Total DNA was extracted from *B. diazoefficiens* from liquid cultures in arabinose-gluconate (AG) medium (39) using the QIAamp DNA minikit (Qiagen). The genomes of LP 3004 and LP 3008 were sequenced using the Illumina HiSeq 2000 platform with 100-bp paired-end reads, achieving a final coverage of 380× and 245×, respectively. The genome of LP USDA 110 was sequenced using the MiSeq platform with 150-bp paired-end reads and 300× coverage.

### Bacterial strains and culture conditions.

The strains used in this study were *B. diazoefficiens* LP USDA 110, LP 3004, and LP 3008 and Escherichia coli DH5α and S17-1 (Bethesda Research Laboratory). *B. diazoefficiens* cells were stored in glycerol stocks at −20°C and in yeast extract-mannitol agar (YMA) (40) at 4°C. For conjugation, peptone-salts-yeast extract (PSY)-arabinose was used (41). If needed, chloramphenicol (Cm), kanamycin (Km), or streptomycin (Sm) was added at a final concentration of 20 mg ml^−1^, 150 mg ml^−1^, and 400 mg ml^−1^, respectively. E. coli strains were grown at 30°C in LB (42). If needed, Km was added at a final concentration of 10 mg ml^−1^. The pK18*mobsacB* plasmid (43) was used for cloning and conjugation.

To analyze flagella in a liquid medium, bacteria were cultured in minimal Götz medium (34), and for swimming motility analysis, bacteria were inoculated with a sterile toothpick onto the same medium containing 0.3% (wt/vol) agar as previously described (12) and were incubated for 10 days.

### Construction of mutant strains.

DNA amplifications were carried out by PCR using *Pfu* polymerase (Productos Bio-Logicos, Buenos Aires, Argentina) in a Biometra TOne thermocycler (Analytik Jena, Jena, Germany). The oligonucleotide primers used in this study were supplied by Genbiotech SRL (Buenos Aires, Argentina). Digestions were achieved using Promega (Biodynamics SRL, Buenos Aires, Argentina) enzymes as necessary. DNA sequencing was performed using a DNA sequencing service (Macrogen Corp., Seoul, South Korea).

The *B. diazoefficiens* LP 3004 Δ*6743-6744* deletion mutant was obtained using the unmarked in-frame deletion strategy as previously described (44). To generate the construction, a 348-bp fragment of the upstream region of blr6743 and a 344-bp fragment downstream of blr6744 were amplified using the primer pairs Up_Fw_6743 (5′-AAA AGA ATT CAT GAT GGT GGC GAT GAA C-3′)/Up_Rv_6743 (5′-GCC GTC GAC GGA TCC GAG GCA GTT CTG CAA GGC CCA GTC-3′) and Dw_Fw_6744 (5′-TGC CTC GGA TCC GTC GAC GGC CAA GAT CGA CGC GGA CTA C-3′)/Dw_Rv_6744 (5′-AAA AAA GCT TGA CCC ATA CCC TTC AAC TGG-3′), respectively. The resultant fragments were purified and combined as the template to be amplified with the primer pair Up_Fw_6743/Dw_Rv_6744. The 713-bp fragment obtained was cloned into the EcoRI/HindIII sites of the pK18*mobsacB* vector to generate pK18*mobsacB*::6743-6744. The gene replacement was made by mobilization of the construction from *E. coli* S17-1 to *B. diazoefficiens* LP 3004 by biparental mating, and the simple crossover (cointegrate) was selected by Km resistance. To induce double-crossover recombination, the selected transconjugants were plated onto yeast mannitol agar (YMA) supplemented with 10% (wt/vol) sucrose. The resulting clones were checked by PCR using primers outside the recombination region, Fw_cheq_6743 (5′-CAA CAT CTT CCC CTC CAA CA-3′)/Rv_cheq_6744 (5′-CGG CGA CGA CTA CCA GAA C-3′), in order to select the mutant genotype, and the correct in-frame deletion was verified by DNA sequencing.

To interchange the allele of LP 3008 with the wild-type one, the fragment Up was amplified with the primer pair Up_Fw_6743/Up_Rv_6743 using LP 3004 as the template for the PCR (Up_wt). The fragment was cloned into the EcoRI/HindIII sites of the pK18*mobsacB* vector to generate pK18*mobsacB*::Up_wt. After that, the procedure was the same as before; in the first selection with Km, the B. *diazoefficiens* LP 3008 genome contained the plasmid and two Up fragments, one wild type and one altered (Up_wt and Up_del, respectively). When the second crossover was selected with 10% (wt/vol) sucrose, the transconjugants could have the fragments Up_wt or Up_del. In order to check which one of the alleles was selected, a PCR employing the primers Up_Fw_6743/Up_Rv_6743 was performed and the product of the reaction was observed in a 2.5% agarose gel because the 11-bp difference was noticeable. The fragments of LP 3004 and LP 3008 were used as controls. If two similar-size fragments appeared (around 348 bp), the plasmid was not excised from the chromosome, and the clone was discarded. The resulting clones were checked by PCR using primers outside the recombination region, Fw_cheq_6743/Dw_Rv _6743, and the correct in-frame deletion was verified by DNA sequencing.

### Flagellin separation and analysis.

The preparation of flagellins was carried out as described previously (13). Briefly, rhizobia grown in liquid medium to an optical density at 500 nm (OD_500_) of 1.0 were vortexed for 5 min and centrifuged at 10,000 × *g* for 30 min at 4°C. The supernatant was collected and incubated with 1.3% (vol/vol) polyethylene glycol 6000 and 166 mM NaCl for 2 h at 4°C. This suspension was centrifuged at 11,000 × *g* for 40 min at 4°C, and the pellet was finally resuspended in phosphate-buffered saline. For analysis, the samples were boiled in Laemmli loading buffer for 10 min and then separated by sodium dodecyl sulfate-polyacrylamide gel electrophoresis (SDS-PAGE) (45). Polypeptide bands were revealed using Coomassie brilliant blue R250.

### Polymorphism analysis.

The raw reads of the three strains were quality filtered using Trimmomatic v0.38 software (46) and assembled using Unicycler software v0.4.7 (47); the resulting contigs were mapped with minimap2 v2.17 (48) to the reference genome. Haploid variant calling was performed using Snippy v3.1 (https://github.com/tseemann/snippy) via its docker image (https://hub.docker.com/r/ummidock/snippy_tseemann). The raw sequences from LP USDA 110, LP 3004, LP 3008, and K USDA 110 were analyzed using Snippy with the DR USDA 110 sequence as the reference. Snippy-core was then used to build a core SNP alignment, which was used to construct a phylogenetic tree with PhyML v3.3.3 software. The K80 DNA evolutionary model was selected, as indicated using ModelTest-NG (49), and nonparametric bootstrap (1,000 replicates) was used for branch support. Snippy software was also applied to identify the SNPs among the strains of this work, using the DR USDA 110 sequence as the reference. Venn diagrams were generated using the Venn tool at http://bioinformatics.psb.ugent.be/webtools/Venn/. A Circos plot was generated using Circos v0.69-8 software (50).

To assess whether an SNP could result in a mutation affecting the protein function, we used the ConSurf server (26) to assign a score from 1 to 9, indicating the degree of conservation of any position in the ortholog family, and a possible functional role.

Additionally, orthologs of the proteins that presented SNPs were found using BLASTP (E value, <10^−4^; query coverage, >70%) to search for associated mutant phenotypes at the Fitness Browser (http://fit.genomics.lbl.gov) database. Searches with no hits were repeated using PSI-blast instead.

### Data availability.

The raw reads of LP USDA 110, LP 3004, and LP 3008 have been deposited in the NCBI Sequence Read Archive (SRA) database and can be accessed under the accession numbers SRR11840083, SRR12102326, and SRR12102325, respectively.
